# Rewiring tumor cytokine networks to enhance immune checkpoint blockade: mechanisms, engineering, and clinical translation

**DOI:** 10.1186/s13046-026-03656-z

**Published:** 2026-02-07

**Authors:** Xiaodong Wang, Junjie Wang, Qianqian Wang, Gouping Ding, Yiping Huang, Yeqian Feng

**Affiliations:** 1 Department of Oncology, The Second Xiangya Hospital, Central South University, Changsha, Hunan 410011 China; 2 Department of Oncology, Zhuzhou Hospital Affiliated to Xiangya School of Medicine, Central South University, Zhuzhou, China

**Keywords:** Cytokines, Tumor microenvironment, Immune checkpoint blockade, Immunocytokines, Oncolytic viruses

## Abstract

Immune checkpoint inhibitors (ICIs) targeting PD-1/PD-L1 and CTLA-4 can produce durable remissions in multiple solid tumors, yet primary and acquired resistance remain frequent, often sustained by an immunosuppressive tumor microenvironment (TME). Cytokines orchestrate immune activation and suppression in the TME, and their crosstalk with checkpoint pathways shapes T-cell priming, trafficking, effector function, and adaptive feedback such as PD-L1 induction. Here we integrate mechanistic evidence with translational strategies to modulate cytokine networks as rational partners for ICIs. We discuss cytokine agonism (native cytokines and engineered variants), targeted immunocytokines and receptor-selective designs, and localized delivery platforms including intratumoral electroporation, viral and gene-therapy vectors. We also review approaches that neutralize immunosuppressive mediators (e.g., IL-6, TGF-β, IL-1β) and cytokine-like pathways that govern trafficking and angiogenesis (e.g., CXCR4 and VEGF). Across these modalities, emerging clinical trial and regulatory experience illustrates both setbacks and tractable successes, highlighting the importance of dose, schedule, and compartmentalization to expand the therapeutic window. Finally, we outline practical principles for next-generation combinations, emphasizing biomarker-guided patient selection, toxicity mitigation, and systems-level modeling to identify nonredundant intervention points. These advances aim to convert “cold” tumors into inflamed lesions and extend durable benefit to broader patient populations.

**Graphical Abstract:**

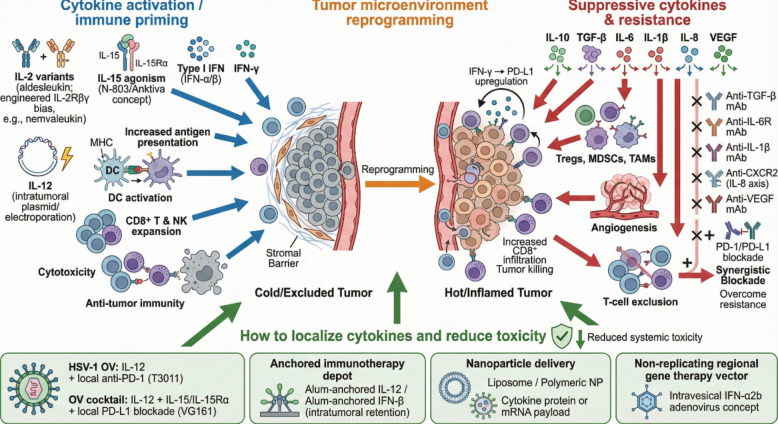

## Introduction

Immune checkpoint blockade (ICB) using antibodies targeting PD-1, PD-L1, and CTLA-4 has achieved remarkable clinical success in multiple solid tumors, offering durable remissions in a subset of patients [1, 2]. However, many tumors display primary non-responsiveness or develop acquired resistance to immune checkpoint inhibitors (ICIs), often because an immunosuppressive tumor microenvironment (TME) impairs T-cell function and limits immune-cell infiltration [3]. Cytokines are a diverse class of intercellular signaling proteins, including interleukins, interferons, tumor necrosis factors (TNFs), chemokines, and growth factors, that orchestrate immune regulation and inflammation [4]. Within the TME, the balance between immune-stimulatory and immunosuppressive cytokines is a central determinant of anti-tumor immunity and of the depth and durability of responses to ICIs [5, 6].

Within the TME, dysregulated cytokine balance can sustain chronic inflammation, angiogenesis, and immune evasion, thereby promoting tumor progression [5]. Conversely, selected pro-inflammatory cytokines can amplify anti-tumor immunity. IL-2, IL-12, IL-15, and type I/II interferons enhance activation, expansion, and cytotoxic programming of cytotoxic T lymphocytes (CTLs) and natural killer (NK) cells [5, 7]. In contrast, immunosuppressive cytokines such as IL-10, TGF-β, IL-4, and IL-6 blunt effector lymphocyte function and recruit regulatory T cells (Tregs), myeloid-derived suppressor cells (MDSCs), and tumor-associated macrophages (TAMs), which collectively suppress anti-tumor immunity [8, 9].

**Fig. 1:**
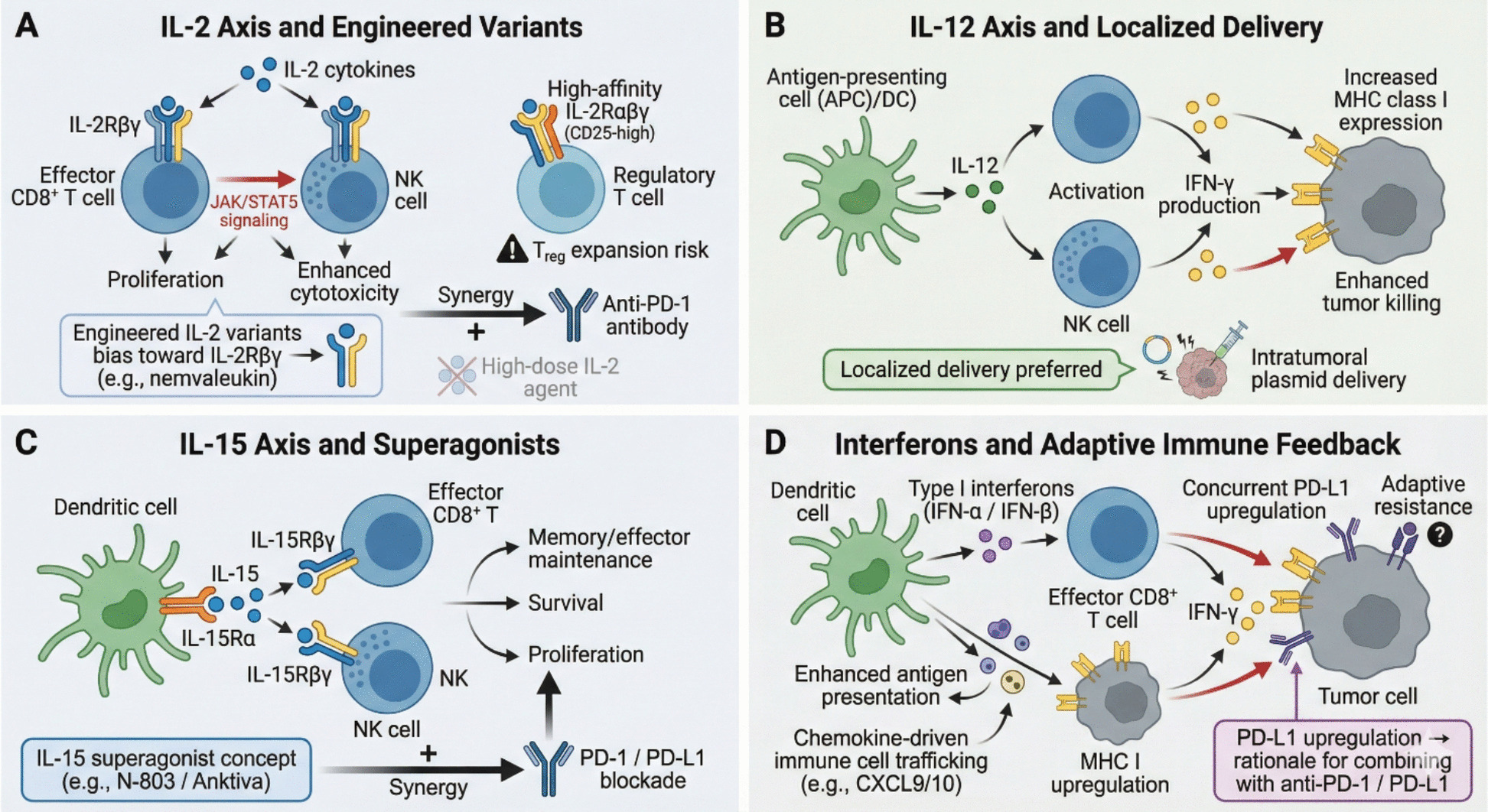
Cytokine axes shaping antitumor immunity and their therapeutic engineering. This figure illustrates key cytokine pathways that promote antitumor immune responses and their therapeutic exploitation. **A** The IL-2 axis activates effector CD8⁺ T cells and NK cells via IL-2Rβγ–mediated JAK/STAT5 signaling, enhancing proliferation and cytotoxicity, while high-affinity IL-2Rαβγ signaling on regulatory T cells (Tregs) poses a risk of Treg expansion; engineered IL-2 variants biased toward IL-2Rβγ aim to preferentially stimulate effector cells and synergize with PD-1 blockade. **B** The IL-12 axis links antigen-presenting cells to NK and T cells, driving IFN-γ production, MHC class I upregulation, and enhanced tumor cell killing, with localized or intratumoral delivery strategies favored to limit systemic toxicity. **C** The IL-15 axis supports NK cell and CD8⁺ T-cell survival, proliferation, and memory maintenance through IL-15Rα–mediated trans-presentation, and IL-15 superagonists further potentiate these effects in combination with immune checkpoint blockade. **D** Type I interferons enhance antigen presentation, immune cell trafficking, and effector CD8⁺ T-cell function, while simultaneously inducing adaptive resistance through PD-L1 upregulation, providing a rationale for combination with PD-1/PD-L1 inhibitors

These cytokine networks intersect with checkpoint pathways. IFN-γ can induce PD-L1 on tumor and immune cells as an adaptive negative-feedback response, while chronic inflammation mediated by IL-6 or IL-1 promotes checkpoint upregulation and T-cell dysfunction [10, 11]. Conversely, engagement of inhibitory receptors such as PD-1 dampens cytokine production, reinforcing exhaustion programs and stabilizing an immune-suppressive TME [12, 13]. These observations support cytokine modulation to shift the balance toward immune activation and thereby enhance ICI efficacy. These reciprocal effects underscore why combining cytokine modulation with checkpoint blockade may convert non-inflamed or suppressed tumors into settings permissive for effective T-cell activity, while anticipating adaptive feedback such as PD-L1 induction [14–16] (Fig. 1).

Historically, high-dose cytokines such as IL-2 (aldesleukin) and IFN-α produced occasional durable remissions in melanoma and renal cell carcinoma, but severe toxicities and limited efficacy restricted use, and ICIs replaced them in many settings [17, 18]. Recent advances in protein engineering and drug delivery have revived cytokines as ICI adjuncts, with the goal of preserving immune stimulation while reducing systemic toxicity [19, 20]. Immunocytokines, including cytokine–antibody fusions and receptor-selective cytokine variants, aim to improve tumor targeting and reduce toxicity; targeted delivery approaches such as cytokine gene therapy vectors further broaden this space, and inhibition of immunosuppressive cytokines (for example IL-6 or TGF-β) has emerged to counter tumor-induced immune suppression and improve ICI outcomes [21–23].

This review surveys cytokine control of anti-tumor immunity under ICI therapy, integrating mechanistic insights with therapeutic strategies, recent clinical trials, and regulatory developments. We discuss challenges in toxicity management and patient selection, and outline future directions for integrating cytokine modulation into immuno-oncology to overcome resistance and improve patient outcomes overall.

## Mechanistic roles of cytokines in ICI therapy

### Pro-inflammatory cytokines enhancing anti-tumor immunity

#### Interleukin-2 (IL-2)

IL-2 is a canonical T-cell growth factor and among the earliest cytokines deployed in cancer therapy. It signals through a trimeric IL-2 receptor (IL-2Rαβγ) [24]. Effector T cells and NK cells predominantly express the intermediate-affinity IL-2Rβγ dimer, whereas Tregs and activated T cells express the high-affinity IL-2Rαβγ complex [25]. Ligand engagement activates JAK1/JAK3 and downstream STAT5, MAPK, and PI3K/Akt/mTOR pathways, driving T-cell proliferation, survival, and differentiation [26]. Functionally, IL-2 robustly expands CD8 + T cells and NK cells and enhances cytotoxicity; however, it simultaneously expands immunosuppressive Tregs, creating a fundamental therapeutic duality [27, 28]. High-dose IL-2 (aldesleukin) can induce tumor regression in melanoma and renal cell carcinoma, but its clinical utility has been limited by severe systemic toxicities (including capillary leak syndrome and cytokine storm) and by Treg expansion that constrains durable effector immunity [27, 29]. These liabilities have motivated efforts to optimize IL-2, particularly in combination with ICIs to reinvigorate T cells after PD-1/CTLA-4 blockade.

To improve IL-2’s therapeutic index, multiple engineered variants and delivery strategies have been developed [30]. A central concept is receptor bias toward IL-2Rβγ to preferentially activate CD8 + T and NK cells while reducing IL-2Rα (CD25) engagement on Tregs [31]. Bempegaldesleukin (BEMPEG; NKTR-214), a PEGylated IL-2 prodrug designed to favor IL-2Rβγ activation, showed early immune activation and an objective response rate of approximately 59% when combined with nivolumab [32, 33]. Nevertheless, a phase III study of BEMPEG plus nivolumab in advanced melanoma failed to improve outcomes over nivolumab monotherapy, prompting discontinuation of development and underscoring that receptor bias alone may not fully overcome IL-2’s clinical constraints [34, 35]. In contrast, newer IL-2 variants such as nemvaleukin alfa—an IL-2/IL-15 mutein that preferentially stimulates IL-2Rβγ—have shown activity even in ICI-pretreated melanoma and renal cell carcinoma and have advanced to phase III trials [36]. Additional optimization strategies include fusion to antibodies or Fc domains to extend half-life and alter biodistribution, together with careful dose and schedule modulation [31, 37]. Determining the most effective combination of receptor bias, pharmacokinetic control, and localized delivery remains a key focus for maximizing IL-2’s anti-tumor efficacy while limiting toxicity.

#### Interleukin-12 (IL-12)

IL-12 is a heterodimeric cytokine (p35 and p40 subunits) produced by antigen-presenting cells that potently induces Th1 immunity [38]. It activates NK cells and CD8 + T cells to secrete IFN-γ, enhancing cytotoxic function and inhibiting tumor growth [38]. IL-12 also promotes CD4 + T-cell differentiation toward the Th1 lineage while suppressing Th2 polarization and, through IFN-γ, upregulates MHC class I on tumor cells to strengthen antigen presentation [39]. Despite strong mechanistic rationale, early systemic IL-12 trials achieved limited efficacy but caused severe inflammatory toxicities, including life-threatening cytokine release syndrome, which curtailed systemic administration and redirected development toward localized delivery approaches [38, 40].

Preclinical studies demonstrate that IL-12 can synergize with ICIs, converting “cold” tumors into inflamed, ICI-responsive lesions [38]. Clinical strategies therefore emphasize intratumoral or locoregional delivery to concentrate IL-12 activity in the TME while minimizing systemic exposure [41]. Intratumoral IL-12 gene therapy delivered via electroporation (tavokinogene telseplasmid), frequently combined with pembrolizumab, has yielded encouraging response rates in melanoma and received breakthrough designation [41]. Ongoing trials are evaluating IL-12-based combinations with ICIs across melanoma, hepatocellular carcinoma, and other malignancies [38, 41]. The guiding principle is that local IL-12 can ignite intratumoral T-cell inflammation and thereby sensitize tumors to subsequent checkpoint blockade [38]. Additional delivery platforms—including oncolytic viruses encoding IL-12 and biomaterials engineered to retain IL-12 within tumors—aim to preserve IL-12’s potent immune activation while avoiding systemic toxicity [38, 42].

#### Interleukin-15 (IL-15)

IL-15 shares common γ-chain signaling with IL-2 yet uniquely supports survival of memory CD8 + T cells and NK cells without expanding Tregs [43]. It promotes homeostatic proliferation of memory T cells and is critical for NK-cell development and function [43]. Within the TME, IL-15 can bolster anti-tumor immunity by sustaining tissue-resident memory T cells and activating NK cells to kill tumor cells [43, 44]. However, IL-15’s short half-life and its requirement for trans-presentation by IL-15Rα complicate systemic therapeutic deployment [44].

To overcome these limitations, modified IL-15 “superagonists” have been engineered. N-803 (nogapendekin alfa inbakicept; Anktiva), a fusion of IL-15 with the IL-15Rα sushi domain that forms a hyper-stable complex, effectively mimics trans-presentation and markedly amplifies NK and CD8 + T-cell activity [45, 46]. N-803 was recently approved by the U.S. FDA in combination with BCG for BCG-unresponsive non–muscle-invasive bladder cancer after achieving a 71% complete response rate in carcinoma in situ, representing the first IL-15-based oncology approval and validating the concept that IL-15-driven expansion of effector lymphocytes can confer meaningful clinical benefit [47]. In ICI combinations, IL-15 is being explored to enhance responses in immune-poor tumors and in patients progressing on checkpoint blockade [43, 44]. IL-15 superagonists and other derivatives are under investigation across solid tumors, frequently paired with PD-1/PD-L1 blockade, with the goal of expanding tumor-infiltrating T and NK cells in situ [43, 44]. Early trials indicate that IL-15 analogs can increase intratumoral lymphocyte proliferation and occasionally mediate tumor regressions, particularly in immunogenic malignancies such as bladder cancer and melanoma; ongoing studies will define optimal dosing, safety, and synergy with ICIs [48, 49].

#### Interferons (type I and II)

Interferons comprise type I IFNs (IFN-α/β) and type II IFN (IFN-γ) and exert potent immunomodulatory and anti-proliferative effects. IFN-α was historically used as adjuvant therapy in melanoma and renal cancer and demonstrated that cytokine-induced tumor control is possible, although its clinical role has diminished with the advent of more effective and tolerable treatments [50, 51]. Type I IFNs are produced by dendritic cells and other cells after sensing pathogens or tumor-derived nucleic acids; they enhance antigen presentation, promote Th1 polarization, and exert direct anti-proliferative effects on tumor cells [52]. In the context of ICI therapy, type I IFNs generated within the TME can “prime” tumors by upregulating MHC molecules and increasing tumor visibility to T cells. For example, radiotherapy and STING agonists can induce local type I IFN and have improved responsiveness to PD-1 blockade in preclinical models [53, 54].

IFN-γ is chiefly produced by activated T cells and NK cells and is a key effector cytokine that activates macrophages, increases MHC expression on tumor cells, and inhibits proliferation and angiogenesis. Paradoxically, IFN-γ induces PD-L1 on tumor and stromal cells, providing a mechanism of adaptive resistance during immune attack [55, 56]. In responding patients, IFN-γ-related transcriptional signatures are often elevated, reflecting ongoing Th1 immunity; however, excessive or chronic IFN-γ may contribute to immune exhaustion and PD-L1–mediated resistance. Therapeutically, systemic interferons are constrained by toxicity, motivating strategies that concentrate interferon activity locally, including oncolytic viruses encoding IFNs and injectable interferon gene therapies. Adstiladrin (nadofaragene firadenovec), a non-replicating adenoviral gene therapy delivering IFN-α2b via intravesical administration, was approved in late 2022 for high-risk BCG-unresponsive bladder cancer and achieved a 51% complete response rate in carcinoma in situ [57, 58]. This exemplifies how high local interferon concentration can be achieved with limited systemic exposure. Interferons also remain critical endogenous mediators of ICI response and are being evaluated in combination regimens, such as intratumoral IFN-β with anti-PD-1 or systemic interferon formulations and interferon inducers paired with checkpoint blockade [59, 60]. Overall, interferons are necessary for effective anti-tumor immunity but require careful modulation to avoid toxicity and immunosuppressive feedback.

#### Tumor necrosis factor-α (TNF-α)

TNF-α is produced by macrophages, T cells, and NK cells and has complex, context-dependent roles in cancer [61]. Acutely, TNF-α can induce tumor-cell apoptosis and promote dendritic-cell maturation; local TNF-α can also increase endothelial adhesion molecules and facilitate T-cell trafficking into tumors [62]. Conversely, chronic TNF-α within the TME can promote cachexia, angiogenesis, and immune suppression. High TNF-α levels have been associated with immune-related adverse events (irAEs) during ICI therapy, and TNF blockade (for example infliximab) is used to manage severe irAEs [63, 64]. There is interest in transient TNF-α blockade in combination with ICIs to improve tolerability and potentially efficacy, as TNF-α may contribute to Treg activity and T-cell dysfunction within tumors. Trials combining ICIs with TNF inhibitors such as etanercept are ongoing to test whether toxicity can be reduced without compromising anti-tumor activity [63, 65]. In parallel, novel approaches aim to harness TNF signaling locally, including oncolytic viruses and TNF superfamily agonists. TNF-α thus exemplifies the narrow therapeutic window of inflammatory cytokines, requiring context-specific modulation to benefit ICI therapy.

### Immunosuppressive cytokines fostering resistance

#### Interleukin-10 (IL-10)

IL-10 is a key anti-inflammatory cytokine produced by Tregs, certain myeloid cells, and exhausted T cells [2]. In the TME, IL-10 suppresses anti-tumor immunity by inhibiting antigen presentation and dampening pro-inflammatory cytokine production [11]. IL-10 can also stimulate CD8 + T cells under specific conditions, motivating clinical exploration of pegilodecakin, a pegylated recombinant IL-10 [66]. However, results were mixed: a phase III trial of pegilodecakin with chemotherapy in pancreatic cancer was negative and development was halted [67]. Generally, high IL-10 in tumors or circulation correlates with poor ICI responses, reflecting an immunosuppressive milieu [68]. Strategies to modulate IL-10 are less advanced than those targeting other cytokines; IL-10 blockade remains conceptually possible, but IL-10 is more commonly viewed as part of a suppressive network produced by Tregs and tumor-associated myeloid cells that must be countered through broader reprogramming.

#### Transforming growth factor-β (TGF-β)

TGF-β is a master regulator of immune suppression and fibrosis in the TME and is often abundant in immune-excluded “cold” tumors where stromal deposition limits effector T-cell infiltration [69]. High TGF-β activity correlates with poor ICI responsiveness [70]. Accordingly, diverse TGF-β inhibitors are under development, including monoclonal antibodies, receptor kinase inhibitors, and ligand-trapping fusion proteins. Preclinical models consistently show that TGF-β blockade can increase T-cell penetration and synergize with PD-1/PD-L1 blockade [71, 72]. A prominent clinical example is bintrafusp alfa, an anti-PD-L1 antibody fused to a TGF-β trap; although early enthusiasm was tempered by later trial results that fell short of expectations, the approach underscored TGF-β as a tractable barrier in the TME [73]. Ongoing trials of selective antibodies and small molecules, frequently combined with ICIs, increasingly emphasize biomarker-driven patient selection, as tumors with high TGF-β signatures or mesenchymal phenotypes may benefit most [74, 75]. Conceptually, matching dominant cytokine programs to tailored combinations—such as PD-(L)1 blockade plus TGF-β inhibition in TGF-β–rich tumors—offers a rational path to overcome this resistance mechanism.

#### Interleukin-6 (IL-6)

IL-6 is a pleiotropic cytokine that drives cancer-related inflammation and immune suppression. It promotes acute-phase responses and supports tumor growth by activating STAT3 signaling in tumor and stromal cells [76, 77]. Immunologically, IL-6 favors Th17 and neutrophil responses yet can suppress effective anti-tumor immunity by expanding myeloid-derived suppressor cells and skewing T-cell differentiation [77]. Elevated IL-6 has been linked to poorer ICI outcomes and increased toxicity, including associations between baseline IL-6 and irAEs such as checkpoint-induced colitis [78]. Persistent IL-6 can maintain suppressive inflammation even when PD-1 is blocked, implicating IL-6 in checkpoint resistance. Consequently, IL-6 blockade using tocilizumab (anti-IL-6R) or siltuximab (anti-IL-6) is being explored as an immunotherapy adjunct [77, 78]. These agents are already used for inflammatory toxicities (tocilizumab is standard for CAR-T cytokine release syndrome and used off-label in severe checkpoint myocarditis), supporting feasibility [78]. Early-phase trials combining IL-6 inhibitors with ICIs are underway [79, 80]. Retrospective observations suggest patients receiving anti-IL-6 therapy for inflammatory diseases may experience improved outcomes on immunotherapy, but prospective confirmation is needed [78]. IL-6 also emerges as a biomarker: high IL-6 identifies inflammatory, checkpoint-resistant disease, and recent analyses have suggested IL-6 may predict immunotherapy failure in lung cancer, supporting incorporation of IL-6 targeting to broaden ICI benefit, with similar patterns observed for related cytokines like IL-8 [81, 82].

#### Interleukin-8 (CXCL8)

IL-8 is a chemokine that recruits neutrophils and MDSCs and promotes angiogenesis [83]. Many tumors secrete IL-8, and high circulating IL-8 is a robust adverse prognostic marker during ICI therapy, reflecting a myeloid-rich immunosuppressive TME [81]. Importantly, dynamic IL-8 changes correlate with outcomes; in advanced lung cancer, a decrease in IL-8 during therapy was reported as the only cytokine change predictive of improved ICI effectiveness, highlighting IL-8 as a particularly informative biomarker [81, 84]. Therapeutically, blocking IL-8 signaling or its receptors (CXCR1/2) may prevent accumulation of suppressive myeloid cells and enhance checkpoint responses [85]. Several CXCR1/2 inhibitors are being tested with ICIs; early signals suggest reductions in MDSCs and potential improvements in response, with manageable safety profiles [85]. Although IL-8 pathway inhibition could theoretically produce inflammatory or vascular side effects, agents such as navarixin and SX-682 have thus far appeared tolerable [86]. IL-8 therefore provides a compelling framework for patient stratification, where high IL-8 may identify individuals who benefit from adding CXCR1/2 blockade to checkpoint therapy.

#### Interleukin-1β (IL-1β)

IL-1β is frequently upregulated in cancer-associated inflammation and promotes tumor progression by supporting angiogenesis, cancer stemness, and recruitment of suppressive myeloid cells; it also induces IL-6 and other downstream mediators that perpetuate inflammatory cycles [87, 88]. Interest in IL-1β inhibition increased after observations from the CANTOS cardiovascular trial, where canakinumab (anti–IL-1β) was associated with reduced cancer incidence, particularly lung cancer, suggesting that interrupting inflammatory signaling can influence tumor biology [89, 90]. In active cancer therapy, canakinumab has been combined with ICIs to test whether IL-1β neutralization improves outcomes. The phase III CANOPY-1 trial (canakinumab plus pembrolizumab in non-small cell lung cancer) did not meet its primary endpoint, but investigation continues in selected settings, consistent with the hypothesis that IL-1β-driven inflammation sustains immunotherapy resistance in specific tumor contexts [91]. Other approaches, including IL-1 receptor antagonism (anakinra), are also being evaluated in combination with ICIs.

#### VEGF-A and other factors

VEGF-A is a growth factor rather than a classic cytokine, but it functions as a potent immunosuppressive mediator that promotes T-cell exclusion through angiogenesis and can directly inhibit T-cell activity [92]. The clinical success of ICI plus anti-angiogenic combinations exemplifies how targeting non-traditional cytokine-like pathways can remodel the TME. By normalizing tumor vasculature and reducing VEGF-driven immune suppression, these combinations enhance lymphocyte infiltration and have established standards of care in select malignancies; bevacizumab combined with atezolizumab in liver cancer is a prominent example [93, 94]. Beyond VEGF, chemokine axes that regulate immune trafficking are also targeted; CXCL12, which retains T cells in the stroma and limits tumor penetration, is being addressed through CXCR4 inhibitors in trials aimed at enhancing immunotherapy [95]. Collectively, these approaches broaden “cytokine modulation” to include chemokines and growth factors that control immune-cell localization and function, complementing strategies that boost pro-inflammatory cytokines or neutralize suppressive mediators.

## Therapeutic strategies to modulate cytokine networks

### Cytokine agonists and engineered cytokine therapeutics

**Fig. 2:**
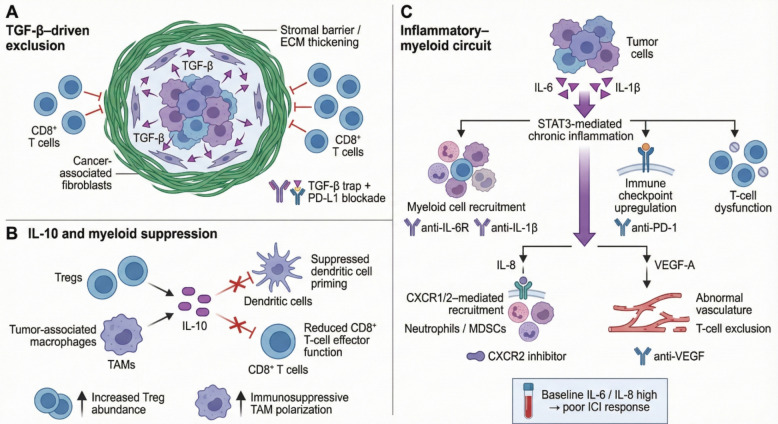
Immunosuppressive cytokine networks driving T-cell exclusion and resistance to immunotherapy. This figure depicts major cytokine-mediated mechanisms that restrain antitumor immunity within the tumor microenvironment. **A** TGF-β signaling promotes stromal remodeling and extracellular matrix thickening, leading to physical exclusion of CD8⁺ T cells from tumor nests; dual targeting of TGF-β and PD-L1 is proposed to overcome this barrier. **B** IL-10 produced by regulatory T cells and tumor-associated macrophages suppresses dendritic cell priming and CD8⁺ T-cell effector function, while fostering immunosuppressive myeloid polarization and increased Treg abundance. **C** Chronic inflammatory circuits driven by tumor-derived IL-6 and IL-1β activate STAT3 signaling, resulting in myeloid cell recruitment, immune checkpoint upregulation, T-cell dysfunction, abnormal angiogenesis, and T-cell exclusion; therapeutic strategies targeting IL-6R, IL-1β, CXCR2, VEGF, or PD-1 aim to disrupt these resistance pathways, particularly in tumors with high baseline IL-6/IL-8 levels

A direct approach is cytokine agonism—using native cytokines at tolerable doses or modified variants—to invigorate T cells and NK cells alongside checkpoint blockade (Fig. 2). High-dose IL-2 (aldesleukin) established proof-of-principle that cytokine-driven immune activation can yield durable remissions, but widespread use declined due to severe systemic toxicity [29, 96]. Contemporary IL-2 engineering aims to preserve effector expansion while minimizing Treg stimulation and cytokine-associated adverse events. Nemvaleukin alfa, an engineered IL-2 variant designed to preferentially activate effector populations, has advanced into late-stage development with the goal of reproducing IL-2’s anti-tumor benefits with an improved safety profile [31, 36]. In parallel, IL-15 agonism has matured from concept to clinical validation: the IL-15 superagonist N-803 achieved FDA approval in 2024 for bladder cancer (in combination with BCG), reinforcing the premise that selective expansion and activation of NK and CD8 + T cells can produce clinically meaningful benefit [46, 49]. These successes have catalyzed broader testing of IL-15–based regimens with ICIs across solid tumors, particularly as a means to expand intratumoral effector pools in PD-1–resistant “cold” tumors.

Beyond IL-2/IL-15, several other cytokines are being revisited as ICI partners. IL-21 promotes CD8 + T-cell function and NK-cell activity; while single-agent studies yielded mixed outcomes, IL-21 is being reintroduced in rational combinations, including regimens pairing IL-21 with nivolumab in melanoma [97, 98]. IL-7 (for example, NT-I7) is being evaluated to support lymphocyte recovery and function during PD-1 therapy, reflecting an emphasis on maintaining immune competence during prolonged treatment [99]. Type I interferons can be administered as PEGylated IFN-α or IFN-β; although their direct anti-tumor effects are generally modest, their ability to enhance antigen presentation and Th1 polarization provides a plausible route to potentiate ICIs in selected settings [100]. Consequently, PEG-IFN-α appears in some combination trials with anti-PD-1 in melanoma. Finally, receptor-targeted agonists in the TNF superfamily are often discussed in this ecosystem because they deliver cytokine-like co-stimulatory signals, even though they are not cytokines per se.

A recurrent theme across agonist programs is that exposure pattern matters as much as cytokine identity. Older paradigms of chronic high-dose infusion frequently triggered systemic inflammation and organ toxicity; modern regimens favor intermittent dosing, careful escalation, or intratumoral administration to induce local immune activation while limiting systemic spillover [38, 101]. Sequential scheduling is also gaining attention. One hypothesis is that initial checkpoint blockade primes or “unlocks” anti-tumor T cells, after which a timed cytokine “boost” (for example, intermittent low-dose IL-2 or IL-15) can sustain memory differentiation and persistence without compounding early toxicity [38, 101]. Early clinical exploration of such sequential or maintenance regimens is underway, aiming to deepen and prolong responses while preserving tolerability.

### Blocking immunosuppressive pathways

A complementary strategy is neutralizing the inhibitory cytokines and mediators that tumors exploit to maintain immune dysfunction. IL-6 blockade (for example, tocilizumab) is motivated by evidence that IL-6 contributes both to resistance and to inflammatory toxicities. Trials are evaluating IL-6/IL-6R antagonists added to ICIs in diseases such as lung cancer and melanoma [78–80]. TGF-β inhibition is being pursued to reverse immune exclusion and stromal barriers, particularly in tumors characterized by fibrotic, T-cell–excluded microenvironments [70]. Multiple modalities—neutralizing antibodies, receptor kinase inhibitors, and bifunctional constructs that sequester TGF-β—are being paired with ICIs in cancers such as liver and urothelial malignancies to facilitate T-cell infiltration [71, 73]. Similarly, IL-8/CXCR2 axis inhibition (for example, SX-682 or navarixin) is being tested with PD-1 blockade in melanoma and pancreatic cancer to reduce MDSC recruitment and improve effector access. IL-1β blockade with canakinumab is being studied in lung cancer across adjuvant and advanced settings, evaluating whether dampening IL-1–driven inflammation lowers recurrence or enhances responsiveness to PD-1 therapy [89, 91]. In parallel, anti-angiogenic strategies targeting VEGF/VEGFR and inhibitors of prostaglandin pathways (including COX2) can be viewed as interventions against immunosuppressive mediators; these combinations have already reshaped practice in renal and liver cancers, illustrating that modifying growth-factor and inflammatory circuits can broaden ICI benefit [94, 102].

A central lesson is that tumors rarely rely on a single suppressive pathway. IL-6, IL-8, TGF-β, and other mediators frequently coexist, forming redundant networks that can compensate when one node is inhibited [5]. This has motivated trials exploring multi-target blockade—such as dual IL-6/IL-8 inhibition—or layered combinations (for example, CXCR2 inhibition plus TGF-β blockade plus PD-1 antibody) in refractory tumors [77]. However, stacking suppressive-pathway inhibitors raises an opposing risk: excessive immune dampening and infection. Consequently, the field is shifting toward “precision immunotherapy,” where the dominant suppressive driver is identified per patient and targeted selectively, rather than broadly suppressing multiple cytokine axes. Biomarker-guided stratification is central to this approach: elevated circulating IL-8 may justify adding a CXCR2 inhibitor, whereas a strong TGF-β transcriptional signature may prioritize a TGF-β trap [70, 84]. Such tailoring aims to maximize therapeutic index—countering the key resistance mechanism while minimizing avoidable toxicity.

### Engineered immunocytokines and fusion proteins

Protein engineering has enabled targeted cytokine delivery and improved pharmacokinetics, offering a route to harness potent cytokines without systemic toxicity [103]. Immunocytokines fuse a cytokine to an antibody or other targeting moiety to concentrate activity within tumors or near selected immune subsets [104]. In principle, attaching IL-2 or IL-12 to tumor-targeting antibodies can amplify local immune activation while sparing normal tissues [41]. Clinical exploration has included an antibody–IL-2 fusion targeting fibroblast activation protein in tumor stroma, as well as constructs using anti-DNA antibodies that localize to necrotic tumor cores [105]. A related and widely used concept is half-life extension via Fc fusion or albumin binding, which increases exposure and can create a “depot-like” effect [106]. IL-15 agonists exemplify this: ALT-803 is built as an IL-15/IL-15Rα–Fc complex to enhance stability and potency [46]. IL-10 has also been engineered as PEGylated forms and as antibody fusions (for example, constructs directing IL-10 to EGFR-expressing tumors) to tune biodistribution [66].

Next-generation designs increasingly combine targeting and function within a single molecule. Conceptually attractive constructs include PD-L1–binding proteins fused to IL-2 or IL-15, which could simultaneously localize to the tumor (via PD-L1 binding and checkpoint blockade) and deliver a cytokine stimulus to nearby T cells [43, 107]. While many such molecules remain preclinical or early clinical, they illustrate a broader design logic: exploit tumor-associated ligands as “addresses” and deliver immune activation at the site of suppression [69].

Nemvaleukin alfa, although not a classical fusion, exemplifies an adjacent strategy: engineering receptor bias rather than anatomical targeting [36]. As a stable monomeric IL-2 variant that preferentially activates IL-2Rβγ, it functions as a systemic cytokine designed to emphasize effector stimulation with reduced toxicity [36]. Nemvaleukin has shown clinical activity both as monotherapy and with pembrolizumab, including responses in subsets of PD-1–refractory patients [36]. If late-stage trials are positive, receptor-biased cytokines may represent a scalable alternative to anatomically targeted immunocytokines, delivering “cell-targeted” rather than “site-targeted” specificity [31, 36].

Overall, engineered cytokines attempt to solve a practical problem: delivering the right immune signal to the right place (or cell type) at the right time. By tuning specificity through targeting domains and controlling exposure through PEGylation or fusion-based half-life extension, these biologics aim to maximize anti-tumor immune activation while limiting collateral inflammation. The field is advancing through iterative learning—both from encouraging signals and from setbacks such as failures of earlier IL-2 prodrugs—which is shaping next-generation designs that more rigorously address stability, receptor engagement, and unintended Treg activation.

### Oncolytic viruses and cytokine gene therapies

Oncolytic viruses (OVs) provide an orthogonal route to cytokine modulation by turning tumors into self-contained inflammatory bioreactors. Engineered viruses selectively infect and lyse tumor cells, releasing tumor antigens and generating danger signals that act as an in situ vaccine. Many OVs are armed with transgenes encoding cytokines or immune modulators, enabling localized payload production in parallel with oncolysis.

The prototypical example is T-VEC, a herpes simplex virus type 1 modified to express GM-CSF, approved for injectable melanoma [108, 109]. T-VEC pioneered combinations with ICIs: pairing with ipilimumab increased response rates in a phase II melanoma study, and combination with pembrolizumab produced durable responses in early-phase testing, although a phase III trial did not meet its primary endpoint [110, 111]. Nonetheless, OV–ICI combinations remain compelling because OVs can inflame tumors and recruit T cells, while ICIs prevent newly primed or recruited lymphocytes from becoming functionally suppressed. Supporting this concept, a study combining a coxsackievirus (CVA21) with pembrolizumab in advanced melanoma reported responses even in PD-1–refractory patients (preliminary phase Ib results) [112].

The field is now moving toward multi-armed OVs that co-deliver cytokines and checkpoint blockade components within a single vector. Two notable herpesvirus-based platforms illustrate this progression. T3011 (MVR-T3011) is an oncolytic HSV-1 engineered to express human IL-12 and a fully human anti-PD-1 antibody, effectively combining intratumoral IL-12 signaling with local PD-1 blockade (phase I/IIa results) [43, 58, 113]. Early-phase trials suggest feasible intratumoral administration and evidence of immune activation consistent with IL-12–driven increases in IFN-γ and cytotoxic programming alongside virus-mediated tumor lysis [38, 43]. VG161 advances the concept further: it encodes IL-12, IL-15, IL-15Rα (to potentiate IL-15 activity), and an Fc-fused PD-L1–blocking peptide, delivering a coordinated inflammatory cocktail plus local checkpoint inhibition [43]. In a first-in-human phase I study in advanced refractory hepatocellular carcinoma, VG161 showed favorable safety with signals of tumor response and TME remodeling, including increased CD8 + T-cell infiltration and cytokine activity within tumors [114]. Such results underscore that complex, multi-payload OVs can be delivered safely and generate measurable immunologic effects in patients.

The rationale for encoding cytokines and checkpoint blockade elements in the same vector is co-localization and synchronization. Viral infection provides innate danger cues and antigen release; IL-12 and IL-15 recruit and expand effector populations; and local PD-1/PD-L1 blockade prevents suppression in the same microenvironment where immune activation occurs [38, 114]. This coordinated, spatially restricted “one-two–three” sequence may be difficult to reproduce with separate systemic agents, and it offers a pathway to inflame “cold” tumors while minimizing systemic toxicity. Beyond these examples, other multi-armed OV programs encode co-stimulatory ligands (such as 4-1BBL or CD40L) or even dual checkpoint inhibitors, reflecting a broader move toward modular OV backbones into which diverse immunomodulatory genes can be inserted to tailor immune outputs [115, 116].

Non-replicating gene therapy vectors provide another clinically validated modality for localized cytokine delivery. Adstiladrin, a replication-deficient adenovirus encoding IFN-α2b administered intravesically, was approved in late 2022 for high-risk BCG-unresponsive non–muscle-invasive bladder cancer [57, 58]. Tavokinogene telseplasmid, an intratumoral IL-12 plasmid delivered with electroporation, has produced complete responses in subsets of melanoma patients when combined with pembrolizumab and has received breakthrough designation [41, 117]. These modalities function as local cytokine factories, achieving intratumoral concentrations that are not feasible with systemic administration. Although such therapies blur the boundary between drugs and procedures, regulatory progress indicates that localized cytokine gene delivery is becoming a practical component of immuno-oncology.

### Clinical advances and approved therapies

**Fig. 3:**
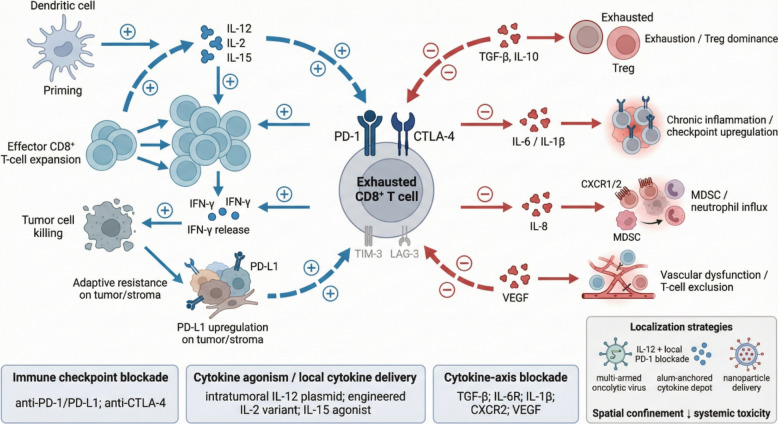
Integrated model of cytokine-driven immune activation, exhaustion, and therapeutic intervention. This schematic summarizes the dynamic balance between cytokine-mediated immune activation and immunosuppressive feedback within the tumor microenvironment. Pro-inflammatory cytokines such as IL-2, IL-12, and IL-15 promote dendritic cell priming, effector CD8⁺ T-cell expansion, IFN-γ release, and tumor cell killing, while also inducing adaptive resistance through PD-L1 upregulation on tumor and stromal cells. Sustained antigen exposure and inhibitory signaling via PD-1, CTLA-4, TIM-3, and LAG-3 drive T-cell exhaustion. In parallel, suppressive cytokines and inflammatory mediators (TGF-β, IL-10, IL-6, IL-1β, IL-8, VEGF) promote Treg dominance, myeloid-derived suppressor cell and neutrophil infiltration, vascular dysfunction, and T-cell exclusion. The lower panel highlights therapeutic strategies to restore antitumor immunity, including immune checkpoint blockade, cytokine agonism or localized cytokine delivery, cytokine-axis blockade, and spatially confined delivery platforms designed to enhance efficacy while minimizing systemic toxicity

While no cytokine–ICI combination has yet been approved as a single packaged regimen, multiple regulatory milestones already validate the broader concept that remodeling the TME can enhance immunotherapy. Several cytokine-based or cytokine-adjacent therapies are now approved and are shaping how clinicians integrate cytokine modulation into standard practice (Table 1) (Fig. 3).

**Table 1:** Representative clinical studies of cytokine-modulation strategies combined with immune checkpoint inhibitors

Cancer type/population	Trial/Reg. No	Combination strategy	Cytokine target/mechanism	Phase/design	Key clinical outcome	Reference
Unresectable/metastatic melanoma (1L)	PIVOT IO 001 (NCT03635983)	Bempegaldesleukin (NKTR-214) + nivolumab vs nivolumab	Engineered IL-2 prodrug intended to bias IL-2Rβγ signaling (reduce Treg stimulation)	Phase III, randomized, open-label	Negative pivotal study; no improvement over nivolumab; program discontinued	Bempegaldesleukin Plus Nivolumab in Untreated Advanced Melanoma: The Open-Label, Phase III PIVOT IO 001 Trial Results (Diab, J Clin Oncol, 2023) [34]
Advanced solid tumors (incl. melanoma, RCC)	ARTISTRY-1 (NCT02799095)	Nemvaleukin alfa ± pembrolizumab	Engineered IL-2/IL-15 mutein designed to preferentially expand effector T/NK cells	Phase I/II, dose-escalation/expansion	Objective responses reported including in ICI-pretreated disease; manageable safety	Nemvaleukin alfa monotherapy in patients with advanced melanoma and renal cell carcinoma: results from the phase 1/2 non-randomized ARTISTRY-1 trial (Calvo, J Immunother Cancer, 2025) [36]
Metastatic melanoma (intratumoral approach)	e.g., NCT03132675	Intratumoral IL-12 plasmid electroporation + pembrolizumab	Local IL-12 expression to inflame tumors and synergize with PD-1 blockade	Phase II (reported)	Encouraging response rates reported with some durable responses	Phase II Trial of IL-12 Plasmid Transfection and PD-1 Blockade in Immunologically Quiescent Melanoma (Algazi, Clin Cancer Res, 2020) [118]
Advanced solid tumors (intratumoral cytokine delivery)	NCT03805919	Tolododekin alfa (anchored IL-12 drug conjugate) ± ICI	Anchored IL-12 retained in tumor to enhance Th1/IFN-gamma responses while limiting systemic exposure	Phase I, first-in-human	Feasible safety profile and immune activation reported; supports combination development	Interleukin-12 anchored drug conjugate (tolododekin alfa) in patients with advanced solid tumours: a phase 1 clinical trial (Kaufman, Nat Commun, 2025) [119]
BCG-unresponsive NMIBC (CIS ± papillary)	QUILT-3.032 (NCT03022825)	N-803 (IL-15 superagonist) + BCG	IL-15 pathway agonism to expand NK and CD8 + T cells and reinforce immune surveillance	Registration-enabling cohorts (reported)	High complete response rates reported in CIS with durable remissions; FDA approval (2024)	IL-15 Superagonist NAI in BCG-Unresponsive Non-Muscle-Invasive Bladder Cancer (Chamie, NEJM Evid, 2023) [49]
BCG-unresponsive NMIBC	Phase III (reported)	Intravesical nadofaragene firadenovec (IFN-alpha2b gene therapy)	Local IFN-alpha2b gene delivery to drive antitumor immunity in bladder	Phase III, single-arm, open-label, repeat-dose	Complete responses achieved with bladder-sparing potential; acceptable safety	Intravesical nadofaragene firadenovec gene therapy for BCG-unresponsive non-muscle-invasive bladder cancer: a single-arm, open-label, repeat-dose clinical trial (Boorjian, Lancet Oncol, 2021) [58]
Advanced melanoma after ICI ± targeted therapy	C-144–01 (NCT02360579)	Lifileucel (autologous TIL) with lymphodepletion + high-dose IL-2 support	Adoptive TIL transfer supported by IL-2 to promote in vivo persistence	Phase II, multicenter	Durable objective responses reported in ICI-refractory melanoma	Efficacy and safety of lifileucel, a one-time autologous tumor-infiltrating lymphocyte (TIL) cell therapy, in patients with advanced melanoma after progression on immune checkpoint inhibitors and targeted therapies: pooled analysis of consecutive cohorts of the C-144–01 study (Chesney, J Immunother Cancer, 2022) [120]
Metastatic melanoma	Randomized phase II	Ipilimumab + sargramostim (GM-CSF) vs ipilimumab	GM-CSF to enhance antigen presentation and myeloid activation alongside CTLA-4 blockade	Phase II, randomized	Overall survival improved and toxicity reduced in combination arm	Ipilimumab Plus Sargramostim vs Ipilimumab Alone for Treatment of Metastatic Melanoma: A Randomized Clinical Trial (Hodi, JAMA, 2014) [121]
Advanced unresectable melanoma	Randomized phase II	T-VEC (GM-CSF oncolytic HSV-1) + ipilimumab vs ipilimumab	Oncolytic virotherapy plus GM-CSF expression to inflame tumors and synergize with CTLA-4 blockade	Phase II, randomized, open-label	Higher objective response rate in combination; long-term follow-up reported	Randomized, Open-Label Phase II Study Evaluating the Efficacy and Safety of Talimogene Laherparepvec in Combination With Ipilimumab Versus Ipilimumab Alone in Patients With Advanced, Unresectable Melanoma (Chesney, J Clin Oncol, 2018) [110]
Advanced melanoma	MITCI (NCT02307149)	Intratumoral V937 (Coxsackievirus A21) + ipilimumab	Oncolytic virus-mediated in situ vaccination + CTLA-4 blockade	Phase 1b, single-arm	Objective responses reported with acceptable safety	Intratumoral oncolytic virus V937 plus ipilimumab in patients with advanced melanoma: the phase 1b MITCI study (Curti, J Immunother Cancer, 2022) [112]
Advanced solid tumors (intratumoral OV)	T3011 (ASCO/JCO suppl 2023)	Oncolytic HSV-1 encoding IL-12 and anti-PD-1 (T3011)	Co-expression of IL-12 and PD-1 blockade within a single viral vector	Phase 1/2a	Early feasibility/activity reported in conference abstract	A phase 1/2a study of T3011, an oncolytic HSV expressing IL-12 and PD-1 antibody, administered via intratumoral (IT) injection as monotherapy in advanced solid tumors (Ji, J Clin Oncol suppl, 2023) [113]
Refractory hepatocellular carcinoma	VG161 (reported 2025)	Oncolytic HSV-1 encoding IL-12, IL-15, IL-15Ralpha, and PD-1/PD-L1 blocker	Multi-cytokine immune stimulation plus intratumoral checkpoint blockade delivered by OV	Phase I	Promising early activity and immune remodeling reported	Oncolytic virus VG161 in refractory hepatocellular carcinoma (Shen, Nature, 2025) [114]
PD-L1-high advanced NSCLC (1L)	INTR@PID Lung 037	Bintrafusp alfa (PD-L1/TGF-beta trap) vs pembrolizumab	Concurrent PD-L1 blockade and TGF-beta neutralization	Phase III, randomized, open-label	Negative study; no improvement over pembrolizumab	Bintrafusp alfa versus pembrolizumab in patients with treatment-naive, PD-L1-high advanced NSCLC: a randomized, open-label, phase 3 trial (Cho, J Thorac Oncol, 2023) [122]
Advanced/metastatic NSCLC (1L)	CANOPY-1 (NCT03631199)	Canakinumab + pembrolizumab + chemotherapy vs placebo + pembrolizumab + chemotherapy	IL-1beta blockade to suppress protumor inflammation and potentially enhance ICI activity	Phase III, randomized, double-blind	Did not meet primary endpoint	Canakinumab Versus Placebo in Combination With First-Line Pembrolizumab Plus Chemotherapy for Advanced NSCLC: Results From the CANOPY-1 Trial (Tan, J Clin Oncol, 2024) [91]
Unresectable hepatocellular carcinoma (1L)	IMbrave150 (updated analysis)	Atezolizumab + bevacizumab vs sorafenib	Anti-VEGF plus PD-L1 blockade to reprogram tumor microenvironment	Phase III, randomized	Established standard-of-care with improved survival outcomes	Updated efficacy and safety data from IMbrave150: Atezolizumab plus bevacizumab vs. sorafenib for unresectable hepatocellular carcinoma (Cheng, J Hepatol, 2022) [123]

This table prioritizes primary clinical evidence (randomized trials, prospective single-arm studies, registration-enabling cohorts, and major conference abstracts) and minimizes review-only citations. Reported outcomes are summarized at a high level and may evolve with longer follow-up

*Abbreviations*: *BCG* Bacillus Calmette-Guerin, *CIS* Carcinoma in situ, *HCC* Hepatocellular carcinoma, *ICI* Immune checkpoint inhibitor, *IFN* Interferon, *IL* Interleukin, *NMIBC* Non-muscle-invasive bladder cancer, *NSCLC* Non-small cell lung cancer, *OV* Oncolytic virus, *RCC* renal cell carcinoma, *TIL* Tumor-infiltrating lymphocyte, *TME* Tumor microenvironment, *T-VEC* Talimogene laherparepvec

First, combining ICIs with anti-angiogenic therapy has become a clear paradigm of TME modulation. Atezolizumab plus bevacizumab for advanced hepatocellular carcinoma, approved in 2020, improved survival versus sorafenib, consistent with mechanisms such as vascular normalization and reduction of VEGF-driven immunosuppression [93, 123]. Similarly, first-line renal cell carcinoma regimens commonly include PD-1/PD-L1 antibodies with tyrosine kinase inhibitors targeting VEGF and related pathways, reinforcing that interrupting tumor-promoted growth-factor signaling can broaden ICI efficacy [94].

Second, cytokine addition to checkpoint blockade has precedent. Ipilimumab plus sargramostim (GM-CSF) was approved in 2015 for metastatic melanoma after improved overall survival compared with ipilimumab alone, plausibly through enhanced antigen presentation and myeloid activation [121]. Although later supplanted by ipilimumab–nivolumab combinations in many settings, this regimen provided early clinical proof that a cytokine can incrementally improve checkpoint therapy [121].

Third, adoptive cell therapy has reintroduced cytokine support into modern regimens. Lifileucel, an autologous tumor-infiltrating lymphocyte (TIL) therapy, requires high-dose IL-2 after infusion to support engraftment and persistence. In February 2024, lifileucel (Amtagvi) received FDA accelerated approval based on phase 2 trial data for unresectable or metastatic melanoma progressing after prior therapy, representing the first cellular therapy approved for a solid tumor [120, 124]. This approval effectively “rehabilitates” IL-2 as a supportive element in contemporary immunotherapy and provides a platform for combination exploration, including studies pairing TIL therapy with anti-PD-1 to further improve persistence or function of transferred lymphocytes [124].

Fourth, IL-15 agonism has achieved a pivotal regulatory milestone. N-803 (Anktiva) plus intravesical BCG was approved in April 2024 for BCG-unresponsive non–muscle-invasive bladder cancer after a 71% complete response rate in carcinoma in situ with durable responses in many patients [47, 49]. Notably, this success occurred in a clinical context where checkpoint inhibitors alone have delivered only modest benefit, suggesting that targeted cytokine-driven expansion of NK and T cells can be decisive. This approval also sets the stage for broader testing of IL-15 agonists with systemic ICIs in other tumor types where increasing intratumoral effector density may convert non-responders to responders [47].

Fifth, interferon gene therapy has entered routine oncology. Adstiladrin, approved in December 2022, delivers IFN-α2b via intravesical adenovirus and achieved a 51% complete response rate in carcinoma in situ, offering a bladder-sparing option [57, 58]. Although approved as monotherapy, its mechanism—high local interferon exposure—provides a strong rationale for combination with systemic PD-1 blockade, particularly in bladder cancer where pembrolizumab is already used for BCG-unresponsive disease [57]. More broadly, Adstiladrin demonstrates that regional cytokine gene therapy can be clinically effective and compatible with systemic immunotherapies.

Finally, multiple engineered IL-2 and cytokine variants are approaching decision points. Nemvaleukin alfa has shown activity in melanoma and renal carcinoma, including in patients previously treated with ICIs, and phase III trials underway in melanoma and ovarian cancer could position it as a next approved cytokine therapy [36, 125]. Additional IL-2 variants and cytokine fusions are advancing in trials, including designs intended to further minimize Treg stimulation and systemic inflammation. Local IL-12 strategies are also nearing critical readouts: intratumoral IL-12 plasmid therapy combined with pembrolizumab has produced response rates around 30% in checkpoint-naïve metastatic melanoma with some complete responses and is now being tested in randomized settings, including an ongoing phase III study in anti–PD-1–refractory melanoma to assess whether IL-12 immunogene therapy can rescue ICI failures [41, 118]. A positive outcome would represent a major milestone and could become the first formal approval of a cytokine-plus-checkpoint combination.

Collectively, these developments point to a consistent trajectory: cytokine-based interventions are increasingly finding clinical niches—either as stand-alone regional therapies, as critical supports for cell therapies, or as rational partners to ICIs. By priming immune infiltration, sustaining effector persistence, or dismantling dominant suppressive circuits, cytokine modulation is moving from experimental adjunct to an integral component of immunotherapy design. As of 2025, at least three FDA-approved therapies incorporate cytokine modulation as a central mechanism, with multiple late-stage trials poised to determine whether cytokine–ICI combinations will formally enter standard-of-care practice following forthcoming phase III readouts.

## Challenges and future directions

### Toxicity and safety considerations in cytokine therapy

Systemic toxicity is the foremost obstacle for cytokine therapeutics. By their nature, cytokines amplify inflammatory pathways that, at effective anti-tumor doses, can approach the threshold of cytokine storm [126, 127]. IL-2 and IL-12 are classic examples: the doses needed for robust immune activation can produce severe adverse events such as vascular leak, hypotension, and organ injury, limiting eligibility and dose intensity [126]. Importantly, toxicity is not restricted to “legacy” cytokines; even engineered analogues can provoke serious immune-related adverse events in combination with ICIs [34]. For example, a pegylated IL-2 variant paired with anti–PD-1 produced unexpected immune-mediated toxicities, reinforcing that next-generation cytokines need built-in safety features to achieve a usable therapeutic window [34].

Several convergent strategies aim to widen this window. Short-acting cytokine variants enable rapid titration and prompt discontinuation if toxicity emerges, reducing cumulative exposure [126]. Conditionally activated cytokines—such as protease-activated pro-cytokines or tumor microenvironment-restricted formats—aim to remain functionally silent systemically and become active predominantly within tumors [128]. Local or regional administration can further reduce systemic exposure. In parallel, dosing paradigms are being refined to avoid overwhelming inflammatory peaks, including step-up dosing, intermittent pulses, and schedule optimization to balance immune activation with tolerability [126, 127].

Advanced delivery approaches increasingly focus cytokine action in tumors while sparing normal tissues. One innovative strategy is to anchor cytokines within the tumor microenvironment using binding motifs that attach to extracellular matrix components or to an injected depot [42, 129]. A notable example uses aluminum hydroxide (alum), a long-standing vaccine adjuvant, as a depot scaffold [129]. IL-12 can be engineered with an alum-binding peptide so that, when injected intratumorally mixed with alum, the cytokine remains sequestered locally [129]. In mouse models, a single alum-anchored IL-12 administration persisted in tumors for weeks, induced robust local IFN-γ, activated T and NK cells, and minimized systemic exposure [42, 129]. This approach improved efficacy compared with free IL-12 and, when paired with systemic anti–PD-1, converted poorly immunogenic tumors into curable disease in a substantial fraction of animals [42, 129, 130]. Anchoring type I interferons has shown similar benefits: alum-anchored IFN-β extended intratumoral retention, improved survival, and produced cures with anti–PD-1 without the weight loss and systemic toxicity observed with unanchored cytokine [129, 131]. This “anchored immunotherapy” paradigm demonstrates that physical localization can separate efficacy from systemic inflammation. The concept was popularized in part by work from groups including Wittrup and Irvine, and it is now moving from preclinical demonstration toward clinical testing [129, 131]. Early clinical translation is underway, including alum-anchored IL-12 (ANK-101), with the broader promise that localized cytokines paired with systemic ICIs could function as potent in situ vaccination [119, 129].

Nanoparticle (NP) delivery systems provide a second major route to improve cytokine pharmacology [132–134]. Liposomes, polymeric nanoparticles, and virus-like particles can encapsulate cytokines or cytokine-encoding nucleic acids, protect payloads from degradation, and release them in a controlled manner [132, 135]. These systems can preferentially accumulate in tumors and can be functionalized to target specific cells. In metastatic ovarian cancer models, IL-12 covalently attached to tumor-targeted liposomal nanoparticles and coated with polymers to enhance retention concentrated in tumor nodules and ascites, drove robust CD8⁺ T-cell accumulation, extended survival compared with free IL-12, and sensitized “cold” tumors to PD-1 blockade with evidence of durable immune memory [132, 135]. Such findings support the premise that tumor-focused carriers can convert cytokine administration from systemically inflammatory to locally immunostimulatory, improving therapeutic index [132, 135]. Additional concepts include lipid nanoparticles delivering mRNA encoding IL-12 or IL-21 so cytokines are produced within the tumor, polymer depots that slowly release IL-2 to support T cells during therapy, and cell-attached “backpacks” that carry cytokines on immune cells for guided transport [135, 136]. Notably, nanotechnology can also be used in the opposite direction—nanoparticles designed to sequester excess inflammatory cytokines have been proposed to mitigate cytokine release syndromes—highlighting the versatility of these platforms [137].

In summary, toxicity is being met with engineering ingenuity. By refining activity profiles and localizing exposure, next-generation cytokine therapeutics aim to decouple efficacy from systemic toxicity, an essential prerequisite for routine adoption of cytokine–ICI combinations.

### Predictive biomarkers and patient selection

Given the diversity of cytokine strategies and their potential risks, biomarker-guided patient selection will be vital. ICIs themselves have only imperfect predictive biomarkers, and cytokine combinations compound the challenge: clinicians must decide not only who needs a checkpoint inhibitor, but which cytokine axis to inhibit or augment. Nevertheless, emerging data suggest practical biomarker classes that can support rational deployment.

#### Cytokine and chemokine signatures in tumors

Profiling the tumor microenvironment (TME) for cytokine and chemokine expression can reveal dominant suppressive programs. High TGF-β activity—reflected in TGF-β gene signatures or stromal SMAD activation—supports selecting PD-(L)1 blockade plus TGF-β inhibition [70, 138]. Elevated IL-8 signaling, particularly in tumors enriched for neutrophils and myeloid-derived suppressor cells, supports prioritizing IL-8/CXCR2 pathway inhibition with anti–PD-1 [84]. Conversely, immune-desert tumors with minimal T-cell infiltration and low IFN-γ may require immune priming (e.g., IL-12 or type I interferons) to induce a state in which ICIs can function [139].

#### Baseline and on-treatment circulating cytokines

Blood measurements provide scalable biomarkers that can be repeated longitudinally. High baseline IL-6 has been associated with poor responses to PD-1 blockade in non–small cell lung cancer and has been proposed as both a predictive marker and a therapeutic target [140, 141]. High baseline IL-8 is prognostic of worse ICI outcomes across multiple cancers, and changes over time may be particularly informative: decreases in IL-8 after ICI initiation have correlated with improved efficacy, whereas persistently elevated IL-8 tracks with poor outcomes [81, 84]. Together, these observations support the idea that baseline levels and early dynamics could identify patients who may benefit from adding cytokine-targeted interventions.

#### Gene expression profiling and immune subtype classification

RNA sequencing and targeted immune panels can classify tumors as inflamed, excluded, or desert and identify dominant pathways such as myeloid suppression, wound-healing/TGF-β stromal programs, or IFN-driven exhausted T-cell states [142, 143]. As these tools are increasingly incorporated into clinical trials, they can guide combination choices: a myeloid-suppression signature may direct IL-8 or IL-1 pathway blockade, whereas an IFN-associated exhaustion signature may support cytokine agonism aimed at restoring effector competence [85].

#### Biomarker-first trial design

Future cytokine–ICI trials should treat biomarkers as primary learning objectives, not exploratory afterthoughts. Rather than measuring dozens of analytes without power, a focused set with strong mechanistic rationale (e.g., IL-8, IL-6, IFN-γ–associated chemokines, TGF-β signatures, and key immune cell populations) can yield interpretable decision thresholds [81, 144]. Enrichment designs can then be deployed if benefit concentrates in biomarker-high subsets; in some settings, trials may stratify prospectively (for example, testing IL-6 blockade primarily in patients with high baseline IL-6) to accelerate learning and improve signal detection [79].

A pragmatic decision framework could resemble a simple decision tree: IFN-γ-high but TGF-β-high tumors may warrant adding a TGF-β trap, persistently IL-8-high states may warrant CXCR2 blockade, and immune-desert tumors may require an inflammatory primer such as IL-12 or type I interferons before checkpoint escalation [84, 138].

In practice, an integrated biomarker-driven approach would combine baseline biopsy-based profiling with blood cytokine monitoring to triage patients to cytokine–ICI combinations that address their dominant suppressive mechanism, while early on-treatment readouts could enable response-adaptive intensification [81]. This personalized framework remains in development, but the necessary components—robust assays, mechanistic rationale, and prospective trial integration—are increasingly in place.

### Overcoming redundancy and optimizing combination design

The immune system contains extensive redundancy and layered feedback, and tumors often exploit multiple suppressive circuits simultaneously. Blocking a single cytokine may therefore have limited impact if parallel pathways compensate—for example, IL-6 neutralization may leave IL-1 or TNF programs intact, and TGF-β blockade may be followed by increased IL-8–mediated recruitment of suppressive myeloid cells [71, 77]. This redundancy likely contributes to the mixed performance of single-agent cytokine inhibitors in solid tumors and argues for strategy-level precision in combination development.

One path forward is rational combinatorial blockade guided by mechanism and biomarkers rather than empirical stacking. Dual inhibition of IL-6 and IL-8 could be advantageous in tumors where both axes drive myeloid suppression, potentially restoring T-cell function more effectively than either alone [77, 80]. Another concept is pairing a cytokine agonist with a suppressive cytokine antagonist—e.g., IL-2 or IL-15 to expand cytotoxic lymphocytes together with TGF-β or IL-10 blockade to remove inhibitory constraints—thereby amplifying effector cells while preventing dominant negative feedback [66, 71].

Timing and sequence relative to checkpoint blockade is pivotal and likely cytokine-specific. Clinically, this implies that multiple schedules may need testing; for example, an intratumoral cytokine could be introduced after a few cycles of anti–PD-1 to exploit early T-cell priming, or conversely used up front to create the inflammation required for PD-1 blockade to function [41, 60]. Some cytokines may function best as “primers” administered before or early during ICI therapy—an intratumoral IL-12 pulse can inflame the TME, increase antigen presentation, and recruit/activate effector cells, after which PD-1 blockade may prevent adaptive exhaustion and sustain function [41, 43]. For other cytokines, maintenance after an initial ICI response may be preferable—low-dose IL-2 or IL-15 can sustain expansion of memory-like T cells and reduce relapse risk once tumor burden declines [44].

Finally, combination design must address feasibility: additive toxicity, dosing hierarchies, and cost. Synergy may allow lower dosing of one component or intermittent cytokine pulsing rather than continuous exposure. Mathematical and computational modeling of cytokine network dynamics, integrated with pharmacodynamic data, may help identify intervention points that maximize nonredundant benefit while minimizing toxicity.

### Broadening cytokine targets and integrating other modalities

Beyond established cytokines, the next wave will likely introduce additional targets. IL-18 is a pro-inflammatory cytokine that activates T and NK cells, yet tumors can induce an IL-18 binding protein that neutralizes it [145]; engineered IL-18 variants that evade this decoy have entered early clinical evaluation and have been reported to drive IFN-γ and show activity in melanoma when combined with PD-1 blockade [146]. Conversely, IL-27 has immunoregulatory properties and can suppress T-cell activity; antagonizing IL-27 has shown synergy with PD-1 blockade in preclinical models, suggesting another route to lift cytokine-mediated restraint [65, 77].

Chemokine and growth-factor pathways that shape immune cell trafficking are also attractive. In desmoplastic tumors such as pancreatic cancer, CXCL12–CXCR4 signaling can contribute to T-cell exclusion and resistance [95]; CXCR4 antagonism increases T-cell infiltration and improves checkpoint efficacy in preclinical systems, and clinical trials combining CXCR4 inhibitors with ICIs are progressing in pancreatic and colorectal cancers [85, 147].

Cytokine modulation also extends beyond soluble drugs into cell- and gene-based therapies. Tumor-infiltrating lymphocyte (TIL) therapy and CAR T strategies often incorporate cytokine support, such as IL-2 administration after TIL infusion [120]. Engineered T cells can be “armored” to secrete cytokines locally upon antigen engagement (TRUCK-like platforms), for example inducible IL-12 or IL-18 secretion to remodel the TME in vivo and enhance bystander immunity [148, 149]. These approaches embed cytokine delivery within the effector cell itself, aiming to concentrate cytokine activity at the tumor site while limiting systemic exposure.

Finally, radiotherapy and targeted therapies can be integrated with cytokine–ICI regimens. Radiation can induce type I interferons and chemokines that promote immune recruitment and may contribute to abscopal effects [54]; triple combinations of radiation, ICI, and cytokine modulation are therefore being explored. Preclinical studies support synergy between radiation and cytokines such as IL-15 or TNF-α in the context of PD-1 blockade [43]. Targeted therapies can also shift the cytokine milieu by inducing tumor stress and immunogenicity; therefore, coordinated regimens that combine tumor-intrinsic pathway inhibition, cytokine modulation, and checkpoint blockade may be required for resistant tumors [16, 77].

Translationally, cytokine therapies are complex biologics, and multi-agent regimens increase manufacturing and trial complexity. If biomarker-guided personalization becomes standard, companion diagnostics and biomarker-enriched approvals may be needed. Nevertheless, the trajectory is toward multi-faceted immunotherapy that can safely convert “cold” tumors into “hot” ones and sustain durable anti-tumor immunity across broader patient populations.

## Conclusion

Cytokine modulation is re-emerging as an essential pillar of cancer immunotherapy that complements immune checkpoints. The field has learned from setbacks—including high-profile failures of some IL-2 programs such as bempegaldesleukin—and from successes such as advances in IL-15 superagonists, the clinical maturation of TIL therapy, and cytokine-armed viral platforms in trials. It is pivoting toward smarter cytokine engineering, safer delivery technologies that localize activity, and biomarker-driven development. Moving forward, success will depend on rational combination: selecting cytokine agonists or antagonists that match the tumor’s immune biology, integrating them with ICIs using optimized schedules and dosing, and leveraging delivery strategies that expand the therapeutic window. If these challenges can be met, cytokine–ICI regimens have the potential to extend durable immunotherapy benefit to a larger fraction of patients and tumor types by enabling potent, context-specific immune activation with acceptable collateral toxicity.

## Data Availability

No datasets were generated or analysed during the current study.
